# Piecing together the puzzle of emotional consciousness

**DOI:** 10.1093/nc/niad005

**Published:** 2023-04-06

**Authors:** Tahnée Engelen, Rocco Mennella

**Affiliations:** Cognitive and Computational Neuroscience Laboratory (LNC2), Inserm U960, Department of Cognitive Studies, École Normale Supérieure, PSL University, 29 rue d’Ulm, Paris 75005, France; Laboratoire sur les Interactions Cognition-Action-Émotion (LICAÉ), UFR-STAPS, Université Paris Nanterre, 200 Avenue de la République, Nanterre 92001, France

**Keywords:** emotion, consciousness, theories of emotional consciousness, neural correlates of consciousness

## Abstract

The search for neural correlates of emotional consciousness has gained momentum in the last decades. Nonetheless, disagreements concerning the mechanisms that determine the experiential qualities of emotional consciousness—the “what is it like” to feel an emotion—as well as on their neural correlates have far-reaching consequences on how researchers study and measure emotion, sometimes leading to seemingly irresolvable impasses. The current paper lays out in a balanced way the viewpoint of both cognitive and precognitive approaches to emotional consciousness on the basis of commonalities and differences between the claims of some relevant theories of emotions. We examine the sufficiency of the existing evidence in support of the proposed theories of emotional consciousness by going through the methodological specificity of the study of emotional consciousness and its unique challenges and highlighting what can and cannot be imported by advances in research on perceptual consciousness. We propose that there are three key experimental contrasts that are each equally necessary in the search for the neural correlates of emotional consciousness and each contrast alone coming with its own limitations. We conclude by acknowledging some of the most promising avenues in the field, which may help go beyond the current limitations and collaboratively piece together the puzzle of emotional consciousness.

## Background and scopes

Understanding the phenomenal dimension of consciousness, the “what it is like” to have a specific conscious experience (Nagel 1974), has long been considered a “hard” scientific problem (Chalmers 1995). In the last decades, neuroscientists have proposed an increasing number of theories of consciousness (see Seth and Bayne 2022 for a review) to try to close (or at least reduce) the “explanatory gap” that we intuitively feel (but see Dennett 2019) between conscious experience and its neural determinants, the so-called neural correlates of consciousness (NCC; Koch et al. 2016). In recent years, the development of NCC theories has run in parallel with, and sometimes inspired, a particularly intense debate in the field of affective neuroscience around what emotions are and how to study them (Anderson and Adolphs 2014, Barrett 2017b, 2017c, LeDoux and Brown 2017, Panksepp et al. 2017, Berridge 2018, Fanselow and Pennington 2018, Adolphs et al. 2019, LeDoux 2019, 2020b, 2021, Mobbs et al. 2019, Taschereau-Dumouchel et al. 2022). In fact, most of the controversies around how to define and measure emotion stem from a disagreement concerning the mechanisms that determine the experiential qualities of emotional consciousness—the “what is it like” to feel an emotion—as well as on their neural correlates.

Despite the uncertainty around definitions and measures of emotion (Dukes et al. 2021), several authors have brought forward theories of the neural correlates of emotional consciousness (from now on NCeC), which—not surprisingly—are often in contradiction with one another. In our opinion, the strength of the conviction by which, for instance, some theories define core concepts (e.g. “emotion”) is at present only partially justified by empirical data, mostly due to the unique methodological challenges in the study of the NCeC, which we will detail in the following sections. As previously pointed out by others (Pessoa 2019), tight definitions can slow down scientific progress by hampering novel research ideas, which violate theoretical boundaries, rather than pushing them forward. This can create “tunnel vision” (e.g. Paré and Quirk 2017), resulting in difficulty in studying the construct of interest in its full complexity, as well as in making comparisons between theories. Finally, this can lead to aprioristically valuing or devaluing specific measures (e.g. self-reports, physiology, and behavior) or fields (e.g. human vs. animal research) in providing insightful information concerning the NCeC (for a discussion, see e.g. Panksepp et al. 2017). By no means do we question the necessity of theory building for proper confirmatory hypothesis testing, as it is evident from the contrastive approach that we propose in the second part of the present work. Nonetheless, we may be missing crucial knowledge about the elements that are needed for deriving hypotheses from theories, namely concept definition, agreement on valid and reliable measures, definition of the relationships between concepts, boundary conditions, auxiliary assumptions, and statistical predictions (see Scheel et al. 2021), which sometimes leads to seemingly irresolvable impasses. The present paper aims at reflecting upon possible strategies to build this knowledge, in order to strengthen the theories of NCeC and move the debate forward. After reviewing the existing main approaches to emotional consciousness, we will try to challenge some of their fundamental assumptions, often by referring to advancements in research on the neural correlates of perceptual consciousness. The main aim of the present paper is to outline what we think are the theoretical and methodological specificities of the study of the NCeC. We argue that some of these limitations are unique to the study of the NCeC; i.e. they are in part not shared with the study of other contents of consciousness. In this spirit, we will discuss the main experimental contrasts to be used in the search for NCeC, as well as their interpretative value and limitations. In the final section, we will reflect upon the possible strategies to strengthen NCeC theories.

### The contemporary debate on the NCeC

The present section does not mean to provide a full picture of the existing theories of emotion (for comprehensive reviews, see Lange et al. 2020 and Moors 2022). We will instead focus on the place of consciousness in what are, in our view, some of the most influential recent perspectives. For the purpose of this paper, theoretical propositions on emotional consciousness will be divided into two approaches, which we call “cognitive” vs. “precognitive” approaches to NCeC. The cognitive vs. precognitive distinction refers here, respectively, to whether emotional consciousness is proposed to require cognitive processing [e.g. working memory (WM)] to arise or to whether it is thought to precede it. Other dichotomies have previously been put forward, such as cognitive vs. perceptual (Block 2019), higher vs. first order (LeDoux and Brown 2017), or access vs. phenomenal consciousness theories (Block 1995, 2005), but we felt that the cognitive vs. precognitive distinction is more generalizable to the different subtheories within each category, as it will be clear in the next sections. Within each approach, we will highlight important differences between theoretical perspectives, without aiming at presenting the full complexity of each author’s theory of emotion. Of note, the topic of the interaction between affect and consciousness, using a comparative science perspective, has also recently been the object of an excellent review, to which we address the interested reader (Paul et al. 2020).

#### Cognitive approach to emotional consciousness

Overall, emotional theories that we include in the “cognitive approach” to NCeC propose that, while humans and other animals share evolutionarily conserved subcortical circuits to respond to threat, face aggression, or seek rewards (LeDoux and Daw 2018), the activation of none of these circuits alone is sufficient to cause a subjective emotional experience as humans conceive it. Theories within this approach agree that, in order for the individual to experience emotion, additional top-down processes supported by neocortical associative areas are needed (e.g. Seth 2013, Smith and Lane 2015, LeDoux and Brown 2017, Barrett 2017c). The cognitive approach does not deny the existence of behavioral and visceral reactions to salient environmental stimuli but does not support a reliable and specific causal relationship between these reactions and an instance of what we call emotion (LeDoux and Brown 2017, Barrett 2017c). Therefore, in this view, the subcortical circuits do not qualify as NCeC.

The precise way through which neocortical areas sustain emotional consciousness varies from one cognitive theory to another. Among the most influential ones, LeDoux’s “higher-order” theory of emotional consciousness (HOTEC; e.g. LeDoux and Pine 2016, LeDoux and Brown 2017, LeDoux and Hofmann 2018, LeDoux 2019, 2020a, 2020b, 2021, Taschereau-Dumouchel et al. 2022) combined the existing higher-order theories of consciousness (e.g. Rosenthal 2005, Brown 2015) with the previous work on fear and threat responses (for a summary, see LeDoux 2012). For the HOTEC, for an emotional experience to arise, higher-order representations (HORs) of the stimulus, as opposed to the first order—sensorial or conceptual ones—need to enter WM, via the brain’s “general networks of cognition”, which include the lateral and medial prefrontal cortex, insular cortex, and posterior parietal cortex. For instance, for experiencing fear, a threatening stimulus (e.g. a snake) needs first to be represented in visual areas (first-order representation) and integrated with long-term semantic memories—stored in medial temporal and other cortical areas—in a common unconscious WM representation (the first HOR). This unconscious HOR can further be re-represented in WM, including autobiographic (self-related) memories (self-HOROR), becoming conscious. Specifically, for this self-HOROR to be an “emotion” (emotional self-HOROR), the consequences of the stimulus-evoked activation of the defensive circuits, including cortical arousal, behavioral, and physiological reactions, need to be included in the representation in WM as well (LeDoux and Brown 2017). The HOTEC proposes that our “emotion schemas,” i.e. what we know about emotions, which are part of the unconscious HORs, refine with experience, rendering the emotional experience more and more differentiated throughout development. Overall, the theory implies that emotions coincide with subjective feelings (i.e. conscious emotional experiences) and therefore, since emotions can never be unconscious, individuals cannot be mistaken about the emotion that they are feeling (LeDoux and Brown 2017). Hence, introspection and subjective reports, despite not being a one-to-one readout of the experience, are the gold standard for studying emotion (LeDoux and Hofmann 2018).

Within the cognitive approach, most authors agree in identifying WM as a pivotal process to maintain feelings active over short time periods in order to guide decisional and goal-directed behavior (Mikels and Reuter-Lorenz 2019). Nonetheless, not every author agrees that HORs in WM are needed for emotional consciousness to arise. For instance, Smith and Lane (2015, 2016), by combining Prinz’s perceptual model of emotional consciousness (Prinz 2004, 2008) with cognitive theories of consciousness based on a “global neuronal workspace (GNW)” model (Dehaene and Naccache 2001, Baars 2005), proposed that the content of first-order representations of our own bodily reactions to the emotional situation can directly become conscious. This happens when it is globally broadcasted across a network of frontoparietal regions, which renders the first-order representations available in WM, to guide goal-directed behavior. More specifically, emotional episodes are proposed to involve a hierarchical, and iterative, sequence of appraisals of salient stimuli/situations’ representations. The hierarchy runs upstream in the brain from automatic appraisals of basic characteristics (i.e. novelty and concern relevance), which primarily involve the amygdala (AMY) and the hippocampus, to more sophisticated ones (i.e. goal congruence, agency, value compatibility, and affect meaning), subtended by temporal and prefrontal cortical regions. This hierarchy of appraisals triggers and continuously refines bodily reactions (together with cognitive changes), which, as in Damasio’s “as-if loops” (Damasio 1994), can sometimes only be represented and not embodied. In this view, emotions depend on our own perception of the abovementioned bodily changes, again in a hierarchical fashion, from the perception of discrete body features (Stage 1; brainstem), to whole body patterns—which produce phenomenologically distinguishable bodily feelings (Stage 2; anterior insula)—and finally to emotional concepts (Stage 3; rostral anterior cingulate cortex), which categorize bodily feelings in an integrative manner (e.g. different bodily feelings can activate the same emotional concept). Unconscious emotional representations in Stages 2/3 are thought to compete to gain access to consciousness and, if selected via the global broadcasting mechanism in frontoparietal areas, are rendered available to guide deliberative thoughts and action. In line with HOTEC, the GNW theory of emotional consciousness proposes that the NCeC are cortical, largely overlap with NCC of other contents of consciousness (Smith et al. 2018b), and rely on shared cognitive mechanisms (WM and attention). However, differently from HOTEC, GNW theory proposes that emotional representations can be both conscious and unconscious and that our own conscious and reportable emotional experience can sometimes not be aligned with our behavioral and physiological emotional reactions, depending on how the probabilistic competition between active emotional concepts is resolved (e.g. one can act angry without feeling angry; Smith et al. 2018a).

The focus on embodiment and on the probabilistic attribution of emotional concepts to bodily changes in specific situations is also at the core of cognitive theories centered around the notion of “predictive coding” (Seth 2013, Seth and Critchley 2013, Barrett 2017b, 2017c). In this view, the brain is specifically conceived as a Bayesian prediction machine (Clark 2013, Seth 2015), which continuously runs and updates an internal model of the body in the world, in order to perform allostasis, i.e. the regulation of the body based on metabolic cost and benefits (Barrett 2017c, see also Damasio 1994, 1998, 1999, Parvizi and Damasio 2001). Internal models contain (i) sensory predictions concerning the most probable next stimulation, (ii) motor predictions relative to the most appropriate actions to take, and (III) visceromotor predictions regarding anticipated consequences for allostasis. The interoception of internal sensations (Craig 2009) is thought to produce the lower dimensional feelings of affect (i.e. the so-called “core affect”)—valence and arousal—that are basic features of consciousness (Barrett 2017c). Crucially, although core affects are probably shared in humans and other animals (Barrett et al. 2007, Barrett 2017b), they are not emotions, nor are they specific to emotional episodes. While receiving sensory information both through exteroception and interoception, the brain computes a prediction error between the model and the actual state of the body in the world. The internal model that has the best fit, i.e. minimizes the prediction error, constitutes our present perception, namely our “experience.” From this constructionist point of view, emotional experiences (i.e. fear) are concepts (internal models like any other), built upon experience, that best explain the present state of the body in the world and direct action (Barrett 2017c). With regard to the neural correlates, the same reported emotion might depend on the activation of different neural correlates under different contexts, as it has been suggested that there is a many-to-many mapping between emotional categories and the combinations of somato-visceral/cognitive reactions and situational patterns they map to (Barrett 2017c). Nonetheless, some authors insist on the importance of the salience network for running an internal model of the body in the world, centered on the anterior insula and on the ACC (Seth 2013), while others propose a more complex interaction among brain intrinsic networks (Barrett and Satpute 2013, Barrett 2017c).

HOTEC, GNW, and predictive processing theories agree that emotional concepts are learned progressively through each individual’s development, ultimately allowing for the attribution of meaning to our experiences in the world (Barrett 2006, 2017c, Lane et al. 2015, LeDoux and Brown 2017). This implies that each person has their own unique emotional experiences, as the way humans mentally represent, perceive, recognize, and express emotions is profoundly shaped by life history, sociocultural influences, and language (Jack et al. 2012, Crivelli and Fridlund 2018, 2019, Barrett et al. 2019, Jackson et al. 2019). Individuals differ in their ability to conceptualize and understand their own affective states and responses—i.e. “emotional awareness” (Lane and Smith 2021). Multiple possible neurocomputational mechanisms (Smith et al. 2019a, 2019b) explain why some individuals might, for instance, be able to feel and report valenced perceptual experiences of physiological changes (e.g. the distress associated with an increase in heart rate) but fail to be aware of and report a specific emotional category (e.g. fear). The ability to “put feelings into words,” reporting separate and detailed emotional experiences—the so-called “emotional differentiation” or “emotional granularity”—has proven adaptive for both psychological and social well-being and contributes to the heterogeneity of emotional experiences and reports (Kashdan et al. 2015). The GNW theory in particular does not exclude that perceptually based appraisals, preconceptual ones, can trigger bodily changes and simply felt emotions in the absence of further cognitive processing or via subcortical stimulation (Smith and Lane 2015, 2016). Nonetheless, it is safe to say that cognitive theories converge in proposing that, while it is not impossible that other animals experience some form of affect, this is hardly comparable to the complex emotional experience available to humans and will hardly be directly measurable (LeDoux and Pine 2016, Barrett 2017b, LeDoux 2021). In fact, it has been proposed that unjustified inference of subjective emotion from objective behavioral and physiological measures has led animal studies to produce scarce results in developing new pharmacological treatments for mental disorders in humans, such as depression and anxiety (LeDoux and Pine 2016, Taschereau-Dumouchel et al. 2022).

#### Precognitive approach to emotional consciousness

The precognitive approach to NCeC claims either that emotions can be conscious in a way that is inaccessible to introspective scrutiny (preconscious) or that they can remain fully unconscious, in both cases having core neural determinants in specific evolutionary old brain structures, most of which are subcortical (Izard 2007b, Adolphs and Anderson 2018, Berridge 2018, Fanselow and Pennington 2018). Indeed, some authors within this approach align with theories identifying two types of consciousness (e.g. Damasio 1994, 1998, Block 1995, Merker 2007): a “phenomenal” (or “core”) consciousness, which corresponds to the experience, the “what it is like” to be in a particular state from our unique first-person perspective, and an “access” (or “extended”) consciousness, which makes contents of experience available for further cognitive elaboration (e.g. subjective report). As two types of consciousness exist, two different NCC are postulated (Block 2005). Notably, it has been proposed that phenomenal consciousness emerged relatively early in evolution, as a core mechanism of integration of bodily signals and environmental objects, to guide action, and therefore strongly depends on the activation of subcortical brain structures, notably brainstem structures, such as the superior colliculus and its connections with the thalamus (Parvizi and Damasio 2001, Merker 2007, 2013, Shine 2022). Participating in the ongoing debate around the phenomenal vs. access distinction (Block 2007, Naccache 2018), several authors within the precognitive approach explicitly adhere to this distinction (Panksepp 1998b, 2007, Izard 2007a, 2007b, Panksepp et al. 2017, Lieberman 2019), proposing that additional subcortical structures specifically determine the content of emotional phenomenal experience (e.g. the periaqueductal gray; Panksepp 1998a, 1998b); other authors propose instead that emotions can be either conscious or unconscious (and not phenomenally preconscious), still largely attributing an important role to subcortical structure for unconscious emotions, for instance to the SC and the pulvinar (Celeghin et al. 2015, Méndez et al. 2022), the AMY (Anderson and Adolphs 2014, Fanselow 2018), and the striatum (Winkielman and Berridge 2004).

Despite these differences concerning the existence of preconscious vs. fully unconscious emotional states, the underlying theory of emotion is largely shared between the authors within the precognitive approach and therefore more easily summarized compared to the cognitive approach. Overall, this approach proposes that the conscious representation that allows humans to produce verbal reports about what they feel (e.g. “I feel angry”) has no special status in defining what an emotion is. In fact, reportable subjective feelings in humans are only one of the components of a functionally organized set of behavioral, physiological, and cognitive responses to environmental challenges, caused by “central generators” or “central states” of emotions (Anderson and Adolphs 2014, Fanselow 2018). Such central generators would (at least in part) map onto dedicated neural systems (Adolphs 2013), mostly subcortical (Panksepp 2007), which have been shaped by evolution. Accordingly, these ancient circuits control the execution of typical physiological and behavioral responses, such as freezing (Fanselow and Lester 1988, Fanselow 1994), “wanting” (Berridge et al. 1989, Treit and Berridge 1990, Berridge and Valenstein 1991), or RAGE (Panksepp 1998a), which have been conserved since they have proven useful to adapt to environmental challenges. This set of physiological and behavioral responses is therefore a measurable and objective indicator of the activation of the central emotion generator. Since, for instance, the direct stimulation of dedicated emotional neural circuits has reinforcing effects on behavior (i.e. animals actively try to prolong or terminate the stimulation), these circuits are inferred to be responsible for a phenomenal form of emotional consciousness (Panksepp 1998a, 1998b), which is more differentiated than a simple core affect experience of something good or bad (as in Barrett 2017b, 2017c). Other authors argue instead that, since such reinforcing effects on behavior, as well as typical emotion-related neural and physiological activations, can be elicited even in response to emotional stimuli that are not consciously detected and in the absence of changes in subjective reports, emotions can be fully unconscious (Winkielman and Berridge 2004, Winkielman et al. 2005, Celeghin et al. 2015, Berridge 2018). Depending on the author, the functional properties of evolutionarily conserved emotional circuits are thought to be partially (Adolphs 2017) or almost entirely (Panksepp 1998a) conserved between humans and other animals. The precognitive approach largely acknowledges that humans can further elaborate, reappraise, and access core emotional reactions to report them verbally. Some authors even made the distinction between “basic emotions”—the evolutionarily conserved emotional responses—and the associations between such emotions and cognitive responses, acquired through learning, called “emotion schemas” (Ekman 1992, Izard 2007a). In this view, animal studies, as well as research on infants (Izard 1991), are often thought to represent the gold standard for understanding the emotion primitives, non-contaminated by subsequent cognitive elaboration (Panksepp et al. 2017, Berridge 2018, Fanselow and Pennington 2018). Concerning animal research, this also stems from the possibility of directly stimulating/interfering with the central brain state with invasive techniques and measuring effects on behavior, as well as provoking strong emotional states, which would be unethical in humans (Panksepp et al. 2017). Overall, it is expected that animal studies will be crucial (and for the precognitive approach, they already have been) for gaining a better understanding of psychopathology and how to treat it (Panksepp et al. 2017, Berridge 2018, Fanselow 2018, Fanselow and Pennington 2018).

### Implications of the disagreements between approaches to studying the NCeC

The two approaches have some important points of disagreement. The primary theoretical point on the nature of emotional consciousness is whether the experience of emotion requires (or not) cognitive elaboration to take place. The “cognitive” approach proposes that it does, either in the form of a hierarchy of higher-order representations in WM (e.g. LeDoux and Brown 2017), or via the competition of first-order representations for the global broadcasting in WM (e.g. Smith and Lane 2015), or through the top-down categorization of bodily states in the world via emotional concepts (e.g. Barrett 2017c). The “precognitive” approach proposes that cognition is not needed, either because the phenomenal preconscious experience is automatically elicited by the activation of central emotion generators in the brain and differs from the ability to reflect upon this experience (access consciousness) (e.g. Ekman 1992, Panksepp 2007, Izard 2007b) or because emotions can be fully unconscious when the stimulation of these circuits is not strong enough to produce conscious emotional experiences (Winkielman and Berridge 2004, Anderson and Adolphs 2014, Fanselow 2018). Notably, this distinction can also be seen from the perspective of whether emotional consciousness and its neural correlates are largely shared with other contents of consciousness and mostly cortical (cognitive approach) or are specific to emotion and mostly subcortical (precognitive approach).

Overall, such disagreements have far-reaching consequences concerning the study of emotion (for recent discussions, see Panksepp et al. 2017, Mobbs et al. 2019, Paul et al. 2020), such as on (i) the neuroimaging techniques that have the capacity of providing evidence for the neural correlates of emotion (non-invasive techniques, such as electro/magnetoencephalography (E/MEG), functional magnetic resonance imaging (fMRI), near-infrared spectroscopy, transcranial magnetic stimulation, transcranial direct current stimulation, and transcranial alternating current stimulation, which have no or limited access to subcortical structures, vs. invasive techniques, such as intracranial EEG, intracranial stimulation and lesion studies, which can access these structures); (ii) the measures that best capture what we mean by emotion (subjective reports vs. behavioral/physiological responses); (iii) the pertinence of developmental studies, studies on patients with extended cognitive deficits, and animal studies; and (iv) the potential for developing treatment for mental disorders, such as depression and anxiety. Due to their extensive implications, the consequences of building theories of emotional consciousness on preliminary evidence can lead to particularly disturbing closed-ended impasses. This is well exemplified when comparing strong statements by authors on both sides, which are in explicit contradiction. For instance, for LeDoux and Hofmann (2018, p. 67), “the most direct way to assess conscious emotional feelings is through verbal self-report.” In the very same year, Adolphs and Anderson (2018) stated that “A science of emotion should, in the first instance, use behavior, cognition, and neurobiology in its vocabulary. It should not be based on self-report of feelings in people” (p. 51). Of note, not all authors who adopt a cognitive vs. precognitive approach to NCeC would fully commit to these statements, and we think that many bridges between approaches exist. To give an example, some of the theories that we assigned to either the cognitive (e.g. GNW; Smith and Lane 2016) or the precognitive (e.g. Winkielman and Berridge 2004, Celeghin et al. 2015) approach, which disagree on the centrality of cognition in emotional consciousness, agree on the other hand on the existence of both conscious and unconscious emotions and do not fully disregard some measures over others. Here, we propose possible ways to cross bridges even further by (i) identifying the methodological specificities of the study of NCeC compared to other NCC, (ii) highlighting the limitations to some of the claims of existing theories of NCeC, and (iii) exploring the methodological solutions for testing specific hypotheses to build theories on a more solid ground in the future.

## Are times mature to build theories of emotional consciousness and its neural correlates?

### Are separate theories of emotional consciousness and its neural correlates needed?

Let us start with a global reflection concerning theories of emotional consciousness. A principle of parsimony would suggest not to bother building separate theories of emotional consciousness and its neural correlates, unless evidence supports the notion that consciously experiencing an emotion differs in some way from other forms of experience, such as seeing red, feeling a fatigued muscle after exercise, or feeling thirsty. Intuition-wise, emotional phenomenal experience might seem to some of us qualitatively different from other conscious experiences, simply because watching our children making their first steps feels different from watching anybody else walking. Following this intuition makes the “hard problem” of consciousness (Chalmers 1995) even harder when it comes to emotional experience, as there seems to be a “something extra” to emotional consciousness that needs to be explained. As we have seen, across approaches, several authors provide an explanation for this “something extra,” arguing, e.g. that it is the activation of a number of so-called “limbic” areas and connections, together with its cognitive, physiological, and behavioral consequences, that is specific to the emergence of the conscious experience of emotion, irrespective of the differences on whether these areas fully qualify as NCeC (Winkielman and Berridge 2004, Anderson and Adolphs 2014, Berridge 2018, Fanselow 2018, LeDoux 2020a). Other authors insist more on the activation of brain structures that allow for our own perception of changes in bodily states in a given situation as the unique mechanism that separates emotional from other forms of consciousness (e.g. Seth 2013, Smith and Lane 2015). Nonetheless, it is noteworthy that other theorists explicitly reject both the idea that there are dedicated emotional circuits in the brain and that feelings coming from the perception of bodily changes in the world are specific to emotion (Barrett 2017b), aligning with theories that renounced at distinguishing emotional from other forms of experience at the mechanistic level (Russell 2003, Moors 2022), thus apparently questioning the necessity of separate theories of NCeC.

Another argument that is brought in favor of a special relationship between emotion and consciousness is that emotions are often thought to be deeply connected with unconscious aspects of our mental life. As we have briefly introduced, some research showed that, even when not consciously perceived, emotional stimuli can elicit physiological responses and bias behavior, similarly (but not identically) to when the stimulus is consciously perceived. In a phenomenon called affective blindsight, cortically blind patients can identify the emotional expressions of faces above chance level but are incapable of doing the same for facial attributes unrelated to the emotional expression, such as identity (Rossion et al. 2000), potentially arguing for a special status for non-conscious emotional perception (Tamietto and de Gelder 2010). In healthy participants, such findings are typically supported by adapting psychophysical paradigms commonly used in the study of visual consciousness, such as backward masking, binocular rivalry, or continuous flash suppression (Kim and Blake 2005). Results from these paradigms, which manipulate conscious access to emotional stimuli, show a “preferential access to awareness” of emotional stimuli. For instance, there is a dominant viewing time for fearful faces in binocular rivalry (Amting et al. 2010) and times at which fearful faces break through binocular suppression are shortened (Yang et al. 2007). Once again, it is noteworthy that these findings are not unchallenged. Methodologically, it has been shown that differences between paradigms in the way stimulus awareness is suppressed influence emotion priming effects (Faivre et al. 2012). Furthermore, some have shown that, when stimulus awareness is not inferred by stimulus duration, but it is based on actual subjects’ report, affective categorization of emotional stimuli is not better than chance in the absence of stimulus awareness (Lähteenmäki et al. 2015) and AMY’s activation does not differ for the presentation of fearful and neutral faces (Pessoa et al. 2006). By considering the reported awareness, it has also been shown that only some physiological systems respond to unaware emotional vs. neutral stimuli in a continuous flash-suppressing paradigm (Tooley et al. 2017). Other authors more generally argued that when fully controlling for stimulus awareness, neither behavioral nor physiological responses to emotional vs. neutral stimuli are observed (for a review, see Tsikandilakis et al. 2021). Mixed findings and opposing conclusions concerning unconscious emotional processing (Mertens and Engelhard 2020, Rohr and Wentura 2021) indicate that these mechanisms are still partially undetermined.

Definitive evidence either in support of or against the necessity of building separate theories of emotional consciousness is still lacking, as the debate on the “something extra” to emotional consciousness, as well as on the specificity of unconscious emotional processing, wages on. However, and most importantly for the scope of the present paper, we will argue that irrespective of one’s opinion on the matter, the study of emotional consciousness comes with unique methodological challenges, which we will further elaborate in the following sections. More than the proven necessity of a separate theory of NCeC, it is the specificity of the methodological challenges to the study of emotional consciousness that justifies, in our opinion, that this research gets its own empirical and theoretical attention before it can be clustered together with the existing theories of consciousness.

### Can content-specific NCeC be separated from neural prerequisites and consequences?

Across both approaches, a number of theories of emotional consciousness were predominantly built on evidence coming from research on single emotions, mostly on fear (e.g. Anderson and Adolphs 2014, Celeghin et al. 2015, LeDoux and Brown 2017, Fanselow 2018), despite this not being the case for all theories (Winkielman and Berridge 2004, Panksepp 2007, Izard 2007b, Seth and Critchley 2013, Smith and Lane 2015, Barrett 2017b). This emphasis on fear research is understandable, as it derives from the robustness and reliability across species of fear/threat-related paradigms, such as fear/threat conditioning (e.g. Büchel and Dolan 2000, Delgado et al. 2006, Fullana et al. 2020). Nonetheless, this solution comes with an important limitation, namely the possibility to make a distinction between the content-specific NCC and the full NCC (Koch 2004, Koch et al. 2016). Content-specific NCC are defined as the neural substrates of specific phenomenal characteristics within an experience, e.g. the unique experience of seeing a face. Thus, the content-specific NCC differ from the so-called full NCC, i.e. the neural substrates of consciousness experience in its entirety, irrespective of the specific content of experience, meaning the combination of content-specific NCC for all possible contents of experience (Koch et al. 2016). To isolate content-specific NCC, typically, in the perceptual consciousness literature, contrastive approaches are used, for instance comparing brain activation when consciously perceiving a face vs. not consciously perceiving it, relying on the experimental paradigms introduced in the previous section. Importantly, a known shortcoming of such a contrastive approach is that it reveals not only content-specific NCC for perceiving a face but also all neural substrates preceding and following conscious perception, the so-called neural prerequisites and neural consequences of consciousness (Aru et al. 2012, de Graaf et al. 2012).

On the one hand, the neural prerequisites of consciousness refer to the mechanisms that are necessary for the conscious experience to arise, as, for instance, in some circumstances, the fact of directing attention toward the stimulus, but are not the determinants of the content of the phenomenal experience (de Graaf et al. 2012). On the other hand, neural consequences are the aftereffects associated with a given phenomenal experience, for instance, when an episodic memory automatically comes to mind after consciously perceiving an object, like the Proustian “madeleine” (de Graaf et al. 2012). One proposed way of separating the different types of NCC relates to the notions of content invariance and content specificity. In more detail, if a neural substrate is involved in the emergence of two distinct phenomenal experiences (content invariance), it cannot explain the subjective difference between the two (content specificity) and thus is more likely to be a prerequisite or a consequence than a content-specific NCC (de Graaf et al. 2012).

We argue that these notions directly apply to the search for NCeC. For instance, it is quite undebated that global affect dimensions such as arousal, valence, and action tendencies participate to different extents in the emergence of each and every one of our emotional experiences (Lang and Bradley 2010), therefore, applying the abovementioned logic, qualifying as neural prerequisites/consequences of emotional experience, rather than as content-specific NCeC. This is in line with what is proposed by predictive coding theories within the cognitive approach (Seth 2013, Seth and Critchley 2013, Barrett 2017b, 2017c), which indeed deny the existence of content-specific NCC of what we call “emotions,” as emotions are learned concepts that vary across individuals and cultures, and therefore show idiosyncratic brain activation in similar emotional circumstances (“in those theories, variability is assumed to be the norm, rather than a nuisance to be explained after the fact”; Barrett 2017a, p. 9). But even if we refer to the theories that admit the existence of specific NCeC, the focus on fear over other emotions might have involuntarily produced a bias over the importance of specific neural structures over others. As an example, the amygdala and its subcortical/cortical connections are at the core of a number of theories in both approaches, either as an important central emotion generator (Anderson and Adolphs 2014, Celeghin et al. 2015, Adolphs and Anderson 2018, Fanselow 2018, Fanselow and Pennington 2018), responsible for both conscious and unconscious fear, or at least as a necessary determinant for qualifying the conscious experience as “emotional” (see “emotional self-HOROR”; LeDoux and Brown 2017). Not surprisingly, and in our opinion partially due to the fear-centered lenses and to the methodological difficulty in distinguishing content-specific NCC from prerequisites/consequences, it is debated whether this AMY-centered network in humans is really fear/threat specific (Méndez-Bértolo et al. 2016, Burra et al. 2019, McFadyen et al. 2019) or it generally encodes affect dimensions such as arousal (Lin et al. 2020), action relevance (Guex et al. 2020), or stimulus valence (Kragel et al. 2021).

Partially supporting the confusion between content-specific NCeC and its prerequisites/consequences, research focused on other phenomenal emotional experiences, such as disgust, has found quite remarkably different neural correlates (e.g. the ventral striatum and the insula; Chapman and Anderson 2012, Berridge 2018). Importantly, a similar confound applies to any research focusing on only one content of experience. Back to disgust and to the involvement of insula and generally interoceptive cortices (Chapman and Anderson 2012), many authors agree that interoception is central in a wide range of emotional experiences (e.g. Zaki et al. 2012, Pavuluri and May 2015), as we draw upon signals coming from our body to understand how we feel, and might for the very same reason as before not fully qualify as NCeC. It has to be noted, to avoid oversimplification, that the idea that the entire range of emotions that we are capable of experiencing would map onto one single brain area or network is refuted by both cognitive and precognitive approaches and irrespective of whether the authors believe emotions to be discrete or continuous in nature. For example, authors neither opposing (Clark-Polner et al. 2016) nor supporting (Saarimäki et al. 2016, 2018) the existence of discrete neural signatures of emotions would argue that all kinds of emotional experience can be mapped onto a single generalized network. It is therefore at odds that approaches to emotional consciousness sometimes overlook the necessity of fully integrating research on different contents of emotional consciousness before theory building. These premises made how to exactly define what constitutes an emotional content is far from being resolved as, for instance, the debate on whether emotions are continuous or discrete in nature is still lively (Celeghin et al. 2017, Barrett 2017a). The way we define an emotional content can directly impact our methods and conclusions we draw from the results. For instance, it has been shown that assigning experimental stimuli to specific emotional categories has an impact on the performance of supervised machine learning algorithms that look for their brain correlates and that the same categories are sometimes not retrieved with non-supervised algorithms (Azari et al. 2020). The need for clarity in content definitions in the search for NCeC, which we will develop further throughout the paper, calls for even more caution when constructing theories of emotional consciousness.

### Are the NCeC cortical or subcortical?

When it comes to the specific claims on NCeC, the cognitive approach argues that, in order for a conscious emotional experience to exist, domain-general cognitive mechanisms, such as WM and attention, need to be recruited. In support of this claim, subcortical activations are proposed to be insufficient for conscious emotional experiences to arise and cortical activations are thought to be needed, namely activation in a prefrontal-parietal network (Smith and Lane 2015) or more precisely in specific regions of the prefrontal cortex (PFC) (Seth 2013, LeDoux and Brown 2017), which has indeed been found to correlate with self-reported emotions (Williams et al. 2006). Criticisms concerning the centrality of the prefrontal cortex in conscious experience have already been raised in the domain of perceptual consciousness, resulting from the findings obtained with the so-called no-report paradigms (Tsuchiya et al. 2015). As the name indicates, these paradigms require no subjective report from participants and rely on the idea that conscious contents can be inferred from physiological and behavioral changes, within specific experimental conditions. For instance, by inferring the perceived direction of competing moving stimuli under binocular rivalry from eye movements, rather than from subjective reports, it has been shown that frontal activations that were present during the perceptual transition in the report task were absent in the no-report variant of the task (Frässle et al. 2014). While the debate on the necessity of the prefrontal cortex in perceptual consciousness continues (Boly et al. 2017, Odegaard et al. 2017, Block 2019, Northoff and Lamme 2020), emotional theories of consciousness claiming for a role of the prefrontal cortex face on this point the same methodological challenges as their non-emotional counterparts.

However, when it comes to the involvement of the prefrontal cortex in the NCeC, we argue that an additional methodological challenge exists. Namely, it is not clear yet how a no-report paradigm could be adapted to an emotional context. In the emotional domain, even if the participant is “aware” of a fearful face or of an attacking snake, one cannot unambiguously assume that they are also “experiencing” a corresponding emotion. A direct example of how no-report paradigms might be challenging to adapt to emotional stimuli comes from the work by Vetter et al. (2019). In their study, the authors presented both angry and fearful faces using a continuous flash suppression paradigm. They showed that, even in the absence of awareness of the face stimulus, eye movements were still influenced in a seemingly goal-directed fashion, notably deviating toward the fearful face and away from the angry one (Vetter et al. 2019). These findings, which align with the results for spontaneous approach/avoidance decision to task-irrelevant emotional faces (Mennella et al. 2020, 2022, Vilarem et al. 2020, Grèzes et al. 2021), show how emotional information can drive adaptive behavior in the absence of conscious experience, challenging a systematic inference of emotional states from behavior. Overall, in light of the possibility that the PFC might not be necessary for the phenomenal experience, but to the capacity to report it, we suggest that rigorous evidence in the support of the necessity of prefrontal areas to form conscious emotional experiences is lacking, in particular because no emotional counterpart to the no-report paradigm has been conceived at present.

It is important to point out that the sufficiency of subcortical structures for the emergence of emotional consciousness, as proposed by the precognitive approach, is equally debated. Possibly due to the abovementioned fear-related bias, a longstanding question in the domain has been whether conscious fear can or cannot be experienced without intact AMYs. The precognitive approach typically refers to lesion studies in which bilateral AMY damage impairs the recognition of fearful faces, the conscious experience of fear, and fear-related avoidance behaviors (Adolphs et al. 1994, Feinstein et al. 2011), as well as AMY’s stimulation studies in humans that elicited in some cases conscious experiences of fear and anxiety (e.g. Lanteaume et al. 2007, Inman et al. 2020). On the other hand, the authors within the cognitive approach more often refer to the studies in which patients with AMY damage were still able to experience conscious fear (Anderson and Phelps 2002, Feinstein et al. 2013, 2016). Mixed findings are not surprising, however, for both methodological and theoretical reasons. Methodologically, irrespective of the value of patient and lesion data in providing causal insight into brain functioning (Adolphs 2016, Vaidya et al. 2019), such experiments come with limitations. Congenital lesions often result in plastic restructuring of the brain (Wieloch and Nikolich 2006), and stimulation studies can be difficult to generalize, as different stimulation parameters (such as polarity and intensity) create varying electric fields in different patients (Selimbeyoglu 2010). Theoretically, and more relevant for the present discussion, fear of exteroceptive threats (e.g. a predator) and of interoceptive ones (e.g. hypercapnic states due to exaggerated CO_2_ levels), despite falling under the same “fear” label, is now known to rely on partially dissociable brain mechanisms, and AMY shows a different involvement in the two types of fear (for a recent review, see Feinstein et al. 2022), which likely explains some of the mixed findings. This exemplifies once more how ambiguity and disagreement in the definition of emotional concepts (e.g. fear) might artificially lead to conflicting and seemingly irreconcilable interpretations of the same research results.

As we have argued throughout this section, we believe that, due to (i) the lack of strong evidence against or in favor of the specificity of emotional vs. non-emotional consciousness, (ii) the present difficulty in disambiguating content-specific NCeC from neural prerequisites/consequences, and (iii) unresolved issues substantiating either the predominantly cortical or subcortical nature of NCeC, there is no clear need of committing to either cognitive or precognitive approaches to emotional consciousness nor to accept (or discard) one theory over another, among the proposed ones. This should, in our opinion, free researchers from some of the abovementioned impasses, which might lead to the exclusion of specific research fields, methods, or techniques in the search for NCeC, pushing them to collaboratively refine concepts and find agreements on methods (see the “Conclusions and future directions” section). In the next section, we discuss three fundamental experimental contrasts that are commonly employed in the search for NCeC, focusing on what can and cannot be inferred from each of them. We hope that this discussion can contribute to a shared methodological ground for future theory building and research.

## Experimental contrasts and what can(not) be inferred

We propose that there are three main experimental contrasts that are crucial for hypothesis testing on the NCeC. These are widely employed in the literature, but what we can conclude from them often remains implicit. We here directly point out the interpretive value of each contrast, the extent to which it can eventually be adapted from the perceptual to the emotional consciousness field, and its specificities (and shortcomings) to the study of the NCeC. We argue that, given the limitations of any of the contrasts taken alone, the combination of each of them is crucial to further our understanding of emotional consciousness and its neural correlates. Of note, for simplicity, our discussion focuses on the study of emotional consciousness in response to external stimuli, but we by no means disregard the fact that emotional responses and subjective experience might be caused by fully internal factors, both cognitive (e.g. thoughts and memories) and physiological (e.g. hormonal changes, physical fatigue, and inflammation) factors.

### Contrast 1: subliminal versus supraliminal perception of the emotional stimulus

The contrast between subliminally and supraliminally perceived stimuli is widely used in the studies of both perceptual and emotional consciousness (Mitchell and Greening 2012), which we already introduced in the previous sections. In paradigms such as masking or binocular rivalry, an emotional stimulus can alternate between being perceived by the participant (supraliminal) or not (subliminal) on a trial-by-trial basis. The report of the participant in these cases, if any is recorded, is whether they perceived the stimulus (in cases of masking or continuous flash suppression) or which stimulus they saw (in case of binocular rivalry, meaning that the non-reported stimulus was suppressed from awareness). These two types of trials are then contrasted to infer which areas were additionally activated by the conscious perception of the stimulus (Kim and Blake 2005, de Graaf et al. 2012). As highlighted earlier, this contrast alone is limited in how much insight it can give into the NCeC in two ways. First, whatever neural correlates emerge from this contrast might not be content specific and could rather be a reflection of a prerequisite or a consequence of emotional consciousness (Aru et al. 2012, de Graaf et al. 2012). Second, and more importantly, the subliminal versus supraliminal perception of the stimulus, albeit emotional, does not provide information about whether a corresponding emotion was felt. On the one hand, awareness of the stimulus and the emotion can be dissociated from one another, as not all emotional stimuli consciously perceived provoke subjective emotional experiences. On the other hand, as previously discussed, unconsciously perceived emotional stimuli might influence emotional perception, responses, and decisions (Tamietto and de Gelder 2010, Celeghin et al. 2015), including judgments of stimulus valence (Anderson et al. 2012), while possibly not conscious emotional feelings (Winkielman et al. 2005, Winkielman and Gogolushko 2018). This aspect is also intimately connected with the notion of the respective timescales of perceptual vs. emotional experiences, which adds another level of complexity. On the one hand, it is still debated whether the perceptual conscious experience of visual stimuli correlates with “early” neural activity, around 200 ms after stimulus presentation, i.e. the visual awareness negativity component of the event-related potentials (ERP), or with “late” ERP components, such as the P3 and late positivity (for a recent review, see Förster et al. 2020). On the other hand, consciousness of emotional stimuli (emotional faces) might relate in a different and specific way to the early and late ERP components and their neural substrates, as compared to neutral stimuli (e.g. Sun et al. 2023). Crucially, this whole literature, which we only briefly mention here, moderately informs regarding the timescale of the emergence of the subjective emotional experience, which seems to us much less investigated. This might be due to multiple factors, not least the fact that the timing of the conscious emotional experience after discrete stimuli is inevitably confounded with the time and the neural activity needed to report it. While some studies did investigate the neural correlates of the transition between emotional vs. neutral conscious states, via repeated emotional stimuli presentation, the time needed for the instantiation of the emotional experience, as well as its specificity compared to mood changes for instance (Eldar et al. 2021), deserves further attention. Overall, these considerations point out some of the unique limitations to the use of the “Subliminal versus supraliminal perception” contrast in the search for NCeC. Therefore, the addition of the following contrasts is necessary.

### Contrast 2: self-reported experienced (felt) vs. not experienced (unfelt/different) emotion

To be able to reveal content-specific correlates of emotional consciousness, conditions in which a stimulus elicits a reportable emotional experience need to be contrasted with conditions in which a stimulus elicits either an alternative or an absence of reportable emotional experience. Unlike the previous contrast, this relies on supraliminal presentation, while varying, e.g. stimulus content. This is a founding method in affective neuroscience, which makes use of countless databases of stimuli of different natures (e.g. pictures, sounds, and imagery scripts) that are validated for their capacity to elicit, on average, different emotional experiences in the perceiver. Let us examine the limitations inherent to this contrast for the study of the NCeC. First, if neural activation is compared following a stimulus inducing one conscious emotional experience (e.g. fear) vs. another (e.g. sadness), one can learn about different correlates of the two emotions but cannot conclude much about what determines their conscious experience (an “unfelt” control condition is lacking). Second, if neural activation is compared following a stimulus inducing one conscious emotional experience (e.g. fear) vs. a neutral state, the neural differences will contain the activity needed for the conscious emotion, but it will be confounded by the difference between stimuli, both sensorial and affective ones, such as arousal and valence (see Gasper et al. 2019 for further discussion on the use of neutral states as a baseline in contrasts). Third, for its interpretation, this contrast relies on self-report, through which participants somehow declare whether they consciously experienced an emotion and which one. Interestingly, a recent large-scale survey among researchers in the field of consciousness revealed that, although generally aware of their possible biases in measuring the content of experience, as well as of the abovementioned influence of self-reporting on neural activation, researchers overall declared subjective reports to be their preferred method to measure consciousness (Francken et al. 2022). It is true that, whereas indeed accuracy of self-report based on metacognition abilities has been shown to be poor (Nisbett and Wilson 1977), both consciousness and emotional consciousness are first-person experiences and might be quite accurately accessible through introspection (Overgaard and Sandberg 2012). Nonetheless, previous research in emotion has highlighted that the validity of self-report is influenced by the way scales are constructed (e.g. dimensional vs. discrete), by where the focus of introspective attention is placed, or by the amount of elapsed time before the experience is captured (Robinson and Clore 2002, Jack and Roepstorff 2003, Overgaard and Sandberg 2012). Furthermore, for the study of emotional consciousness, an additional level of difficulty exists, as a given stimulus might evoke not only a feeling of fear but also feelings of anger, panic, sadness, or no feeling at all. Different emotional states can coexist at the same time, a known phenomenon called “dialecticism” (Bagozzi et al. 1999, Lindquist and Barrett 2008). Therefore, even if participants were immediately asked to indicate how fearful they felt on a continuous scale, we might be missing a great deal of relevant conscious experiences. A participant might even be primed to believe that what they had felt was fear by framing the report measure in a particular way. A previous work has already shown that the mere act of asking a participant to report on their feelings can change both physiological and neural responses to a particular stimulus or task (Creswell et al. 2007, Lieberman et al. 2007, Kassam and Mendes 2013). Recent research advancements are putting forward exciting new solutions to try to overcome some of these limitations. For instance, the use of continuous naturalistic stimuli, such as movies, has the power to profoundly modify experience (Kovarski et al. 2022), including emotion (Saarimäki 2021), which can be measured over time on multiple dimensions, without loss of complexity. Continuous behavioral reports of affective (e.g. Smirnov et al. 2019) or discrete emotional states (e.g. Hudson et al. 2020) from the participant can be used, but this comes with the neural confound associated with self-report. To circumvent this, ratings from independent samples can be collected, or automated emotion-feature extraction can be relied upon (Kragel et al. 2019). While each and every one of these methods comes with limitations (for a detailed review, see Saarimäki 2021), these advancements nicely demonstrate where the additional challenge lies when studying emotional consciousness, as well as the possible future solutions. The presented limitations for inference relative to the second contrast bring us to the third and final contrast.

### Contrast 3: presence/absence of a behavioral/physiological response

As a third point, conditions in which a stimulus elicits a given behavioral/physiological response need to be contrasted with conditions in which a stimulus elicits either an alternative or no behavioral/physiological response. As presented in the first section, the precognitive approach supports the idea that behavioral and physiological responses can be a readout of emotional experience in animals and in humans, which cannot verbally report experience (e.g. infants and patients) and, for some authors, represent the sole readout of phenomenal (as opposed to access) consciousness (Panksepp et al. 2017). For instance, as far as behavior is concerned, it has been proposed that it is possible to infer from animals’ defensive behavior (e.g. freezing vs. vigorous escape attempts) their corresponding emotional experience (e.g. fear vs. panic, respectively) (The Predatory Imminence Continuum Model; Fanselow and Lester 1988, Fanselow et al. 2019, Mobbs et al. 2020). While the tight link between emotion and action is undebated, possible limitations to this kind of model are brought by converging research on defensive behavior in animals (e.g. Vale et al. 2017, Evans et al. 2019) and humans (Rotteveel and Phaf 2004, Schlund et al. 2016, Mennella et al. 2020, Vilarem et al. 2020). Such research supports the notion that defensive behavior is flexible and not stereotyped in response to threatening stimuli, resulting from a complex and still partially unexplored interaction of stimulus-driven reactions (e.g. reflexes and automated reactions) and rapid—sometimes unconscious—goal-directed responses (Moors et al. 2017, LeDoux and Daw 2018, Mendl and Paul 2020). This is corroborated by the fact that goal-directed approach/avoidance behaviors to emotional stimuli can be elicited in the absence of a reportable strategy for action (Mennella et al. 2022) and in the absence of stimulus awareness (Vetter et al. 2019). A similar discourse applies to physiological responses, which are indeed typically well correlated to subjective reports of emotion at the group level (Friedman et al. 2014, Taschereau-Dumouchel et al. 2020) but not always to a great extent at the individual level: early studies on the relationship between behavioral ratings and physiological responses showed that a correlation between zygomatic and corrugator muscle responses with the rated experienced valence reached significance in roughly 50% of the participants, and the correlations between skin conductance responses and experienced arousal reached significance in around 30% of the sample (Lang et al. 1993). Such findings, together with the fact that, as discussed earlier, consciously undetected emotional stimuli can elicit physiological responses, have raised the question of whether subjective experience and physiological responses are subtended by the same brain mechanisms. Using multivoxel pattern analysis, Taschereau-Dumouchel et al. (2020) indeed showed that, despite a significant correlation between reported fear and skin conductance responses at the group level, some areas were differentially involved in the prediction of the two measures. Other recent work demonstrated that when modeling what contributes to subjective ratings that participants give in response to affective images, both physiological responses and neural (interoceptive) markers explain unique parts of the variance observed in self-reports (Engelen et al. 2023). This means that although physiological responses did make a significant unique contribution to self-reports, they did not account for all of the variance observed in such ratings. Overall, there is evidence that specific behavior and physiological responses can be more or less correlated with subjectively reported emotional experiences, and individual differences, as well as the intensity of the emotional situation, have been found to influence the strength of this correlation, which is named “emotional coherence” (Mauss et al. 2005). It is noteworthy that increased emotional coherence has been related to well-being (Brown et al. 2020), which aligns with the fact that emotion-related disorders, such as anxiety and depression, are undoubtedly “mental” disorders (Taschereau-Dumouchel et al. 2022) but also behavioral and physiological ones (American Psychiatric Association 2013). This is why the third contrast is necessary to complement the first two and cannot be disregarded as not pertinent for the search of NCeC.

## Conclusions and future directions

We hope to have defended the idea that researchers should feel unconstrained with respect to the existing approaches to emotional consciousness, which, despite their value in pushing toward the formation of integrated and meaningful pictures of the existing research, at present rely on preliminary findings, which do not allow to arbitrate unambiguously between them. We insist that this state of things is not necessarily specific to the NCeC literature (Oberauer and Lewandowsky 2019). Across many domains in psychology and neuroscience emerges the prevalence of a strong hypothetico-deductive method, which focuses on scientific progress as the repeated empirical test of hypotheses entailed by theories, while putting less attention upon the use of systematic and collective methods to developing theories in the first place (Borsboom et al. 2021). Another possibility is to conceive the relationship between phenomena, data, and theories as a circular one, in which, before getting to the formulation of a complex theory, several steps are taken, starting with the identification of relevant phenomena and going through the initial formulation of preliminary explanatory models (prototheories), involving a small set of general principles that putatively explain the phenomena of interest (Borsboom et al. 2021). Theory construction in this sense builds on a range of non-confirmatory activities, including descriptive research and exploratory experimentation, which are crucial to reach consensus on concept definition and measures’ validity, among the elements that are necessary for theory building (Scheel et al. 2021). Back to the specific complexity of the study of NCeC, as we have detailed, methods cannot always be directly translated from the field of perceptual consciousness to the study of NCeC. For example, no-report paradigms, which have led to new insights in the search for the NCC, cannot at present lend themselves in the same way for studying emotional consciousness. Likewise, the use of subjective reports in emotional consciousness might need further methodological elaboration for the study of NCeC as compared to NCC, due to, among other things, the dialectical nature of emotions. Lastly, behavioral and physiological responses play a possibly privileged role in the search for NCeC, as compared to NCC, as emotional coherence varies profoundly across individuals and situations.

Throughout the paper, we presented what we think are the necessary advancements to arrive to the separation of content-specific NCeC. These pertain to (i) how an emotional content of consciousness is defined and (ii) how the experimental contrasts must be combined to avoid confounding factors to the identification of the NCeC. Concerning concept definition, cooperative work in the form of adversarial collaborations is a way in which the energy of different laboratories (even adhering to different theories) can be combined. An adversarial collaboration is a multi-lab effort in which one prediction stemming from a particular theory is empirically tested. Such a collaboration has already been suggested (Seth and Bayne 2022) and set up in the field of consciousness (Melloni et al. 2021), where predictions stemming from GNW and integrated information theory will be directly compared against one another. Most interestingly, an adversarial collaboration has been successfully applied to the field of emotions as well to investigate how facial mimicry and voluntary facial activation can induce feelings of happiness (Coles et al. 2022). Aside from adversarial collaborations, data-driven approaches are particularly promising as they might play a part in the definition of emotion ontologies (Jack et al. 2018), thereby assisting in coming to a consensus on how to define emotional contents, in a more agnostic manner. For example, research into mapping the semantic space of emotional experience using several self-report measures in response to a wide array of emotionally evocative videos was able to identify 27 distinct categories of emotional experiences (Cowen and Keltner 2017). Data-driven approaches have likewise been used to define the characteristics that define dynamic facial and bodily movements (Jack et al. 2014, de Gelder and Poyo Solanas 2021), as well as bodily sensation maps associated with subjective feelings (Nummenmaa et al. 2014).

Concerning the proposed contrasts, individual efforts focused on combining the three experimental contrasts for uncovering the NCeC can possibly benefit from some methodologies newly introduced to the study of emotion, such as computational models of behavior (Roberts and Hutcherson 2019). These methods might help give a more mechanistic account of the computational involvement of distinct brain areas and possibly in the search for NCeC. For instance, recent work combining high-field fMRI, continuous flash suppression, and drift-diffusion modeling showed how the faster breaking through flash suppression of fearful faces is due to more rapid perceptual evidence accumulation, associated with activity in frontoparietal regions, occipital lobe, and AMY. Activity in other areas, such as the insula and posterior cingulate cortex, was rather correlated with a lower decision boundary (Kalhan 2022). Finally, the synthesis of existing data would make the comparison of all the different contrasts (which are unlikely to be addressed in one single study) feasible. Large-scale meta-analyses have become more and more achievable, thanks to the diffusion of open science practices, with the added advantage of increasing the statistical power and determining the consistency of effects (Yarkoni et al. 2010). Again, in the field of emotional consciousness, such an approach has already been shown to be informative, for instance, to summarize evidence about physiological responses to subliminal negative affective stimuli (van der Ploeg et al. 2017).

To draw a final parallel, in the field of perceptual consciousness, there is likewise still little agreement concerning the best theory of consciousness, but at least there seems to be a tendency toward a consensus on which methods should be employed to study the NCC (Francken et al. 2022). Acknowledging the necessity and strengths of all different methods and paradigms at our disposal to study the NCeC would be a major step forward, as the search for the NCeC is likely to stagnate by being exclusionary.
